# Longitudinal Growth Curve Trajectories of Family Dynamics after Pediatric Traumatic Brain Injury in Mexico

**DOI:** 10.3390/ijerph17228508

**Published:** 2020-11-17

**Authors:** Grace B. McKee, Laiene Olabarrieta-Landa, Paula K. Pérez-Delgadillo, Ricardo Valdivia-Tangarife, Teresita Villaseñor-Cabrera, Daniela Ramos-Usuga, Paul B. Perrin, Juan Carlos Arango-Lasprilla

**Affiliations:** 1 Advanced Fellowship Program in Mental Illness Research and Treatment, Mid-Atlantic Mental Illness Research Education and Clinical Center (MIRECC), Central Virginia Veterans Affairs Health Care System, Richmond, VA 23249, USA; grace.mckee@va.gov; 2 Department of Psychology, Virginia Commonwealth University, Richmond, VA 23284, USA; pperrin@vcu.edu; 3 Departamento de Ciencias de la Salud, Universidad Pública de Navarra, 31006 Pamplona, Spain; laieneolabarrieta@gmail.com; 4 Rusk Rehabilitation at New York University Langone Health, New York, NY 10016, USA; paukaperez@gmail.com; 5 University Center of Health Sciences, Universidad de Guadalajara, Guadalajara 44160, Mexico; ricardovaldiviatangarife@outlook.com; 6 Hospital Civil Fray Antonio Alcalde, Guadalajara 44280, Mexico; tvillasenorc@yahoo.com.mx; 7 Neurosciences Department, University of Guadalajara, Guadalajara 44160, Mexico; 8 Biomedical Research Doctorate Program, University of the Basque Country (UPV/EHU), 48940 Leioa, Spain; danielalucia6372@hotmail.com; 9 BioCruces Bizkaia Health Research Institute, 48903 Barakaldo, Spain; 10 Department of Physical Medicine and Rehabilitation, Virginia Commonwealth University, Richmond, VA 23284, USA; 11 IKERBASQUE, Basque Foundation for Science, 48009 Bilbao, Spain; 12 Department of Cell Biology and Histology, University of the Basque Country (UPV/EHU), 48940 Leioa, Spain

**Keywords:** traumatic brain injury (TBI), pediatric TBI, family functioning, family caregivers, Latin America

## Abstract

Pediatric traumatic brain injury (TBI) represents a serious public health concern. Family members are often caregivers for children with TBI, which can result in a significant strain on familial relationships. Research is needed to examine aspects of family functioning in the context of recovery post-TBI, especially in Latin America, where cultural norms may reinforce caregiving by family members, but where resources for these caregivers may be scarce. This study examined caregiver-reported family satisfaction, communication, cohesion, and flexibility at three time points in the year post-injury for 46 families of a child with TBI in comparison to healthy control families. Families experiencing pediatric TBI were recruited from a large hospital in Guadalajara, Mexico, while healthy controls were recruited from a local educational center. Results from multilevel growth curve models demonstrated that caregivers of children with a TBI reported significantly worse family functioning than controls at each assessment. Families experiencing pediatric TBI were unable to attain the level of functioning of controls during the time span studied, suggesting that these families are likely to experience long-term disruptions in family functioning. The current study highlights the need for family-level intervention programs to target functioning for families affected by pediatric TBI who are at risk for difficulties within a rehabilitation context.

## 1. Introduction

Pediatric traumatic brain injury (TBI) constitutes a serious global health burden affecting more than 3 million children worldwide every year [1]. Trauma, which can result from both abuse and accidents, is the leading cause of death and disability among children and adolescents under age 18 around the world [2]. The global incidence of pediatric TBI ranges largely by region, with most countries reporting a range between 12 and 486 per 100,000 children [1]. Mild TBI, as indicated by a Glasgow Coma Scale (GCS) score greater than or equal to 13, accounts for more than 80% of pediatric TBIs worldwide [1]. Boys older than 3 years of age have been found to be more frequently affected by TBI than girls, and a higher incidence is observed in children ages 0–2 and adolescents between 15–18 years [1].

Generally, pediatric TBI leads to a wide range of physical, cognitive, emotional, and behavioral problems, including fatigue and headaches [3], changes in attention and executive function [4], depression and anxiety [5], and aggressiveness and antisocial patterns of behavior [6]. These short- and long-term TBI-related changes in functioning often result in significant academic, social, and family reintegration difficulties [7]. Pediatric TBI presents as a highly stressful and unexpected event that generally has a large impact on children and their family’s environment, structure, and overall functioning. In the majority of cases, the families of children with TBI are not well-prepared to face and/or adapt to the unexpected challenges of their new situation. Moreover, the sequelae of TBI in children frequently result in an increased need for short- and long-term support, as well as constant supervision generally provided by a close family member (e.g., parents, siblings, grandparents). Continued caregiving and supervision of children with TBI have been associated with symptoms of burden and mental health problems in caregivers over time [7,8]. 

The prognosis of pediatric TBI has been associated with multiple factors, some of which are directly related to the injury (e.g., localization, severity), while others indirectly relate to the event (e.g., social support network, access to resources); yet all of these factors interconnect to create a unique pattern of recovery for each child. In recent years, factors indirectly related to the injury, such as family structure and dynamics, have proven to be significantly salient in the recovery process, particularly due to their potential for adaptability and malleability, thus becoming an additional area of focus in the rehabilitation process. Thus, the role of the family, as well as overall family functioning have been more actively investigated and held as an important aspect in the rehabilitation trajectory of children with TBI. In this context, Babikian et al. [7] found that families of children with TBI evidenced worse family functioning when compared to families of children with orthopedic injuries, and among the different degrees of severity, families of children with severe TBI evidenced worse family functioning when compared to mild or moderate TBI [7]. Some studies have found improvement in family functioning over time, while others revealed worsening of family functioning [7]. 

Premorbid and post-TBI family functioning has been identified as an important aspect of TBI recovery [9,10]. Family functioning has been found to have both main and moderating effects on children’s recovery, either by buffering or exacerbating the impact of TBI, especially those related to the child’s functioning and behavior [8,11]. For instance, children from families with a high degree of dysfunction tend to exhibit more behavior and executive function problems when compared to children from functional families [11,12]. Certainly, behavior problems contribute to family stress [13], and it appears that parental communication and psychological functioning predict externalizing symptoms in children with TBI [7,14], while parental psychiatric disorders foreshadow internalizing symptoms in children [15]. As shown in the literature, there is a reciprocal and complex relationship between a child’s recovery and the degree of family adaptability; that is to say, the sequelae of TBI in children affects the family environment as much as the family environment affects the child’s recovery process. 

As Rashid et al. [16] indicate in their systematic review, the vast majority of research aimed at evaluating the overall impact of pediatric TBI on family functioning utilized the Family Assessment Device (FAD), which assesses the following dimensions of family functioning: problem solving, communication, roles, affective responsiveness, affective involvement, behavior control, and general functioning. However, the literature on other important aspects of family functioning, including family cohesion and adaptability, following pediatric TBI is still very limited. For example, Youngblut et al. [17] explored factors that contribute to the perception of adaptability and family cohesion in the parents of children with TBI two weeks after hospital discharge. The results revealed that perception varies among mothers and fathers. Mothers perceived greater family cohesion if their psychological stress at the time of admission was low and if they perceived high social support at the time of discharge. Moreover, mothers with a larger number of children, more financial worry, and more stress about the medical team’s behavior at the time of the hospital admission identified greater family adaptability two weeks post-discharge, which the authors attributed to a possible generalization of the often chaotic hospital environment to the home. Similar to mothers, fathers’ perception of family cohesion was dependent upon a greater level of social support at the time of discharge. However, in contrast to mothers, fathers’ perception of family adaptability was not related to stress, social support, their mental health, or the severity of the child’s injury.

In a follow-up study, Youngblut et al. [18] explored which factors affected the perception of adaptability and family cohesion in the mothers of children with TBI 3 months post-discharge. The results of this second study were somewhat similar to the authors’ initial findings. Greater perception of family adaptability 3 months post-TBI was dependent upon greater social support at the time of discharge, as well as on lower levels of stress experienced by the mother while the child was hospitalized. Regarding the perception of family cohesion, mothers identified greater cohesion 3 months post-discharge when their psychological stress was low at the time of the hospital admission and if both parents lived in the same household. 

Despite this growing body of research, there is currently a lack of research regarding family functioning in pediatric TBI from culturally diverse families [16]. In Latin America, for instance, only a small number of research studies have explored adaptability and family cohesion following TBI in adult populations, while studies exploring these same factors in children are nonexistent. For example, in a sample of adults with TBI in Mexico, Lehan et al. [19] found that the more positive the perception of adaptability and family cohesion was for patients and their caregivers, the more positive their perception about communication and family satisfaction was. In a different study by Perrin et al. [20] with a group of caregivers of adults with TBI in Mexico, the authors found that low overload and greater overall life satisfaction led to greater family satisfaction and cohesion. 

Cultural aspects can contribute to the framework in which families operate and face the impact of pediatric TBI. In Latin America, and more specifically in Mexico, family is a cornerstone of society. Families are generally close, and there is an inherent duty to care for sick family members, particularly those with a disability (“familismo” [21]). Further, Latin American families tend to be larger in comparison to North American or European families, and extended family members (e.g., second cousins) are typically considered a close part of the family unit. In essence, a family within Latino cultures functions as a broad support system in many aspects including economic, functional, physical, and emotional support. As such, and given the nonexistent research exploring the experiences of Latin American families facing pediatric TBI, the present study seeks to compare several aspects of family functioning among the families of children with TBI over the course of one-year post-injury to same-age healthy controls in Mexico.

## 2. Method

### 2.1. Participants

Forty-six children with TBI and 46 matched healthy controls were recruited from Guadalajara, Mexico. The sociodemographic characteristics of the sample are presented in Table 1. Inclusion criteria for TBI patients were: (1) being between 7 and 17 years old at the time of injury; (2) have a diagnosis of TBI confirmed by clinical history or laboratory tests (i.e., MRI, etc.) at the time of the evaluation; (3) have a Glasgow Coma Scale score ≤ 15; (4) do not have visual, auditory, or sensorial problems; (5) no history of neurological alterations, developmental disorders, learning disabilities, psychiatric disorders, and substance and/or alcohol consumption; (6) not having an intellectual disability; and (7) having reading and writing proficiency in Spanish.

Inclusion criteria for control group participants were: (1) being between 7 and 17 years old at the time of the first evaluation; (2) not having visual, auditory, or sensorial problems; (3) no history of TBI or other neurological alterations; (4) no history of developmental disorders, learning disabilities, or psychiatric disorders; and (5) no history of substance and/or alcohol abuse. Caregivers of children in both groups (TBI and control) had to be healthy, with reading and writing proficiency in Spanish, without history of the following problems: (1) neurological alterations, including TBI; (2) developmental or psychiatric disorders; (3) learning or intellectual disabilities; (4) substance and/or alcohol abuse history; (5) visual, auditory, or sensory problems. 

Nearly half of the injuries were classified as moderate (*n* = 21, 45.7%), with approximately one-third classified as severe (*n* = 15, 32.6%) and the remainder as mild (*n* = 10, 21.7%). The average Glasgow Coma Scale score at 3 months post-TBI was 10.26 (*SD* = 2.75, range = 5–15). The majority of TBIs were sustained by a fall (*n* = 27, 58.7%), with a substantial minority sustained through car accidents (*n* = 16, 34.8%), and the remainder caused by violence/gunshot (*n* = 1, 2.2%), sports injury (*n* = 1, 2.2%), or another unspecified cause (*n* = 1, 2.2%). Thirty-five of the children with TBI experienced loss of consciousness (*n* = 35, 76.1%).

### 2.2. Measures

#### 2.2.1. Sociodemographic Information Questionnaire

A researcher-created questionnaire was used to collect information about gender, age, school grade, parental education level (in years), as well as TBI-related information (i.e., Glasgow Coma Scale, TBI severity, and TBI cause). 

#### 2.2.2. Family Adaptability and Cohesion Evaluation Scales—IV (FACES-IV)

The FACES-IV [22] is a self-report questionnaire used to assess family cohesion, flexibility, communication, and satisfaction. This instrument, developed from the Marital and Family Systems Circumflex model [23], consists of 62 items divided into the Balanced Scales (balanced cohesion and balanced flexibility), the Unbalanced Scales (enmeshed, disengaged, rigid, and chaotic), the Family Communication Scale, and the Family Satisfaction Scale. 

The current study focused on the Family Satisfaction Scale, Family Communication Scale, and two Balance Scales (Cohesion and Flexibility). Cohesion refers to the extent to which family members report feeling an emotional bond with one another, while flexibility refers to the family’s ability to adapt appropriately to stressors or change. Each item is answered using a 5-point Likert scale, with possible answers ranging from 1 (“Strongly disagree”) to 5 (“Strongly agree”), and responses within each subscale are summed to form a total subscale score. Scores for the Family Communication and Satisfaction scales range from 10–50, and scores for the Cohesion and Flexibility scales range from 7–35. For all subscales, higher scores reflect higher functioning in that domain.

### 2.3. Procedure

All subjects gave their informed consent for inclusion before they participated in the study. The study was conducted in accordance with the Declaration of Helsinki, and the protocol was approved by the ethics committee of the Fray Antonio Alcalde Hospital of Guadalajara, Mexico (HCG/CI-0102/13). The medical history of children admitted to the neuropediatric and neurosurgery units with a TBI diagnosis was reviewed to select children who met inclusion criteria. Parents of children meeting criteria were contacted and invited to participate, and those who accepted were contacted for the first assessment 3 months following the child’s TBI diagnosis. After informed consent was given and signed, children completed a comprehensive neuropsychological battery to measure different domains of cognitive function (e.g., intelligence, attention, memory, processing speed, and executive functioning), while parents provided sociodemographic information and answered questionnaires regarding family dynamics. These measures were collected again at 6 and 12 months following the diagnostic.

On the other hand, participants from the control group were recruited from the Miguel Hidalgo Educational Center. The teachers at the center identified children who matched as closely as possible with the TBI group in sociodemographic characteristics (i.e., age, gender, socioeconomic status, and education level), and who met the study’s inclusion criteria. The teachers provided the researchers a list of names and contact information for these potential control children’s parents, whom the researchers then contacted and invited to be a part of the study. As in the case of the TBI group, parents and children who met the inclusion criteria and who consented to participate in the study were contacted for the evaluations at 3, 6, and 12 months.

Descriptive statistics and *p*-values from group demographic difference tests are presented in Table 1. Paired-samples *t*-tests and chi-square tests showed that children with TBI did not significantly differ from their matched controls on age or sex, but had fewer years of education (*t*(45) = −2.03, *p* = 0.049; 4.59 vs. 5.02 years on average). Parents of healthy control children were more likely to be male (χ^2^ = 5.00, *p* = 0.045) and had more years of education than did parents of children with a TBI (*t*(45) = −8.69, *p* < 0.001). In addition, parents of healthy control children tended to report higher monthly income brackets than did parents of children with a TBI (χ^2^ = 27.41, *p* < 0.001).

#### 2.3.1. Dropout

Fifteen of the 46 children with TBI and their caregivers were recruited near the end of the study, and due to limitations in study administration resources, those subjects were only able to be followed at 3 and 6 months. Thus, data were not available for those 15 families or for their matched healthy control families. However, independent samples *t*-tests showed that families whose data were not available at 12 months did not differ from the other families on average family functioning scores for either the TBI or control groups, *p* > 0.05.

#### 2.3.2. Analytical Plan

In order to account for the interdependent nature of the data, multilevel growth curve models [24,25] were estimated using IBM SPSS 25 (SPSS Inc., Chicago, IL, USA). All models were initially estimated using parameters for the intercept, linear time, and quadratic time only, without parameters reflecting group differences. Quadratic time parameters were retained for the final models if removing them would significantly detract from fit, according to the -2LL goodness-of-fit test [24]. Once the fit of time parameters was established, interaction effects for group differences were entered into the model. Thus, a quadratic effect that did not significantly improve model fit would be removed from the final model, which would then only contain parameters for the intercept, linear time, and group differences at intercept and linear time. 

For all four measures of family dynamics, a linear time model demonstrated the best fit to the data, as a quadratic effect for time did not significantly improve the model and was subsequently removed. Parameters representing group differences in scores at the intercept and for the linear effect of time were then added to the final models. The effect of time was centered at each assessment point, in order to test group differences in scores at each time point. The results presented reflect those from the models centered at the third time point, although model fit and linear effects of time were identical across centering methods within each outcome variable. Further, random effects for intercept and linear time were unable to be estimated for each model and were therefore omitted. Full information maximum likelihood estimation was used to account for missing data at the 12-month follow up. 

## 3. Results

**Figure 1:**
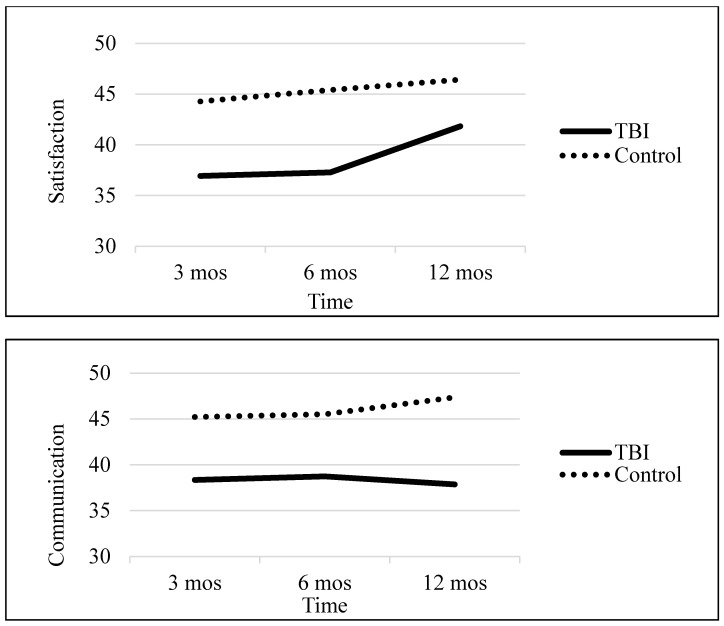

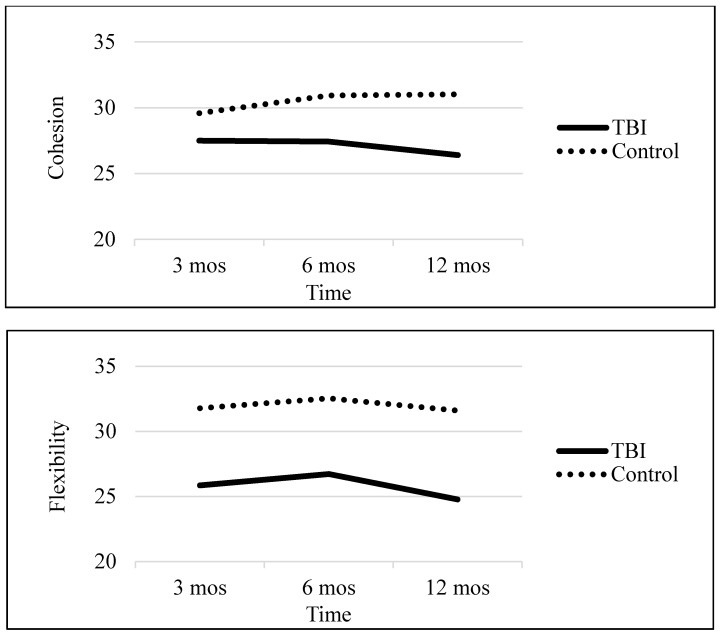
Graphs of Family Functioning Measures over Time by Group.

Descriptive statistics are presented in Table 2. For all measures of family functioning, caregivers of children with TBI reported significantly poorer functioning at each time point, *p* < 0.05, than did control group caregivers (see Table 3 for results from each model and Figure 1 for graphs of family functioning measures over time).

Satisfaction. Results showed that satisfaction scores increased linearly over time, and rates of change did not differ between groups. Taken together, these results suggest that caregivers of children with TBI did not catch up to control group caregivers in terms of satisfaction during the time period assessed; instead, TBI caregivers’ scores remained consistently lower than control caregivers’ scores at each time point.

Communication. On average, family communication did not change over time. However, there was a group by time effect which suggested that the groups differed in rate of change, with communication scores showing no improvement for the TBI group and showing slight improvement for the control group. 

Cohesion. The linear effect for time was nonsignificant, suggesting that, on average, family cohesion did not change over time. However, a significant group by time effect showed that the rate of change differed by group, with cohesion tending to increase slightly for the control group, and actually decreasing slightly for caregivers of children with TBI.

Flexibility. Overall, family flexibility did not change over time. In addition, there were no significant group differences in rate of change over time, showing that caregivers of children with TBI did not catch up at any point to control group caregivers in ratings of family flexibility.

## 4. Discussion

Although previous studies have examined trajectories of family functioning among adults with TBI [26], the current study is among the first to examine family functioning over the course of one-year post-injury reported by parents of children with TBI in Mexico. The study is notable, in that it included multiple time-point data from parents of healthy control children who were matched as closely as possible on age, sex, socioeconomic status (SES), and education level. The results supported the hypotheses that facets of family functioning, including satisfaction, communication, cohesion, and flexibility were significantly lower in families of children with TBI, compared to matched healthy controls at 3 months post-injury. These results are consistent with previous findings demonstrating impaired family functioning in these groups [7]. Importantly, however, these effects persisted for the year following the accident in successive assessments at 6 and 12 months, suggesting that families of children with TBI experience decreased functioning that is sustained for at least one-year post-injury. This study is among the first to demonstrate that, rather than returning to baseline over time, as might be expected of a family experiencing a rupture like that of pediatric TBI, family functioning did not improve over the entire first year post-injury.

Parents of children with TBI reported significantly lower family satisfaction in the year following the child’s injury in comparison to parents of children without a TBI. The results showed that family satisfaction increased linearly for both groups, who did not differ in the rate of change in satisfaction over time. These findings suggest that families of children with TBI experience lower satisfaction with family relationships than do families of children without TBI, and that this difference remains consistent in the year following TBI. Families may experience a drop in satisfaction with family relationships after a child experiences a TBI and may not be able to regain a premorbid level of functioning, at least during the year afterward. Troublingly, the similar rates of change in satisfaction across groups indicate that families of children with TBI are not able to “catch up” with healthy families during this year, and the effects may even persist beyond the first year.

Parents of children with TBI also reported worse family communication and cohesion than did parents of healthy control children. Both communication and cohesion did not appear to increase over time when averaged across the groups. However, for both measures, the rates of change did differ between the groups. In examining the average scores of each group at each time point, families of children with a TBI appeared not to change in levels of communication and decreased slightly in levels of cohesion, while healthy control families appeared to increase slightly in levels of both communication and cohesion. These results provide evidence that families experience difficulties in communication and cohesion in the year after. Even more importantly, the trajectories found here suggest that these difficulties in cohesion may in fact get worse over the first year, although a longer follow-up is needed to empirically establish this effect.

Finally, the families of children with TBI also reported lower family flexibility in the year following injury than controls. Flexibility did not change over time for either group, and there were no differences in rate of change. These results show that families of children with TBI experience difficulties in flexibility and adaptability, such that they may have trouble appropriately adjusting family behaviors or roles in response to stressors or changes, in comparison to healthy control families. Moreover, in the year after injury, these families do not appear to regain the levels of flexibility reported by families of children without TBI.

This study is among the first known to examine family functioning in pediatric TBI across multiple time points in the year following injury, to include a healthy control cohort, and to study this issue in a sample of families in Latin America. These results show that families of children with a TBI appear to experience deficits across multiple aspects of family functioning including satisfaction, communication, cohesion, and flexibility in the year following TBI, in comparison to the families of children without TBI, and that they are unable to overcome these deficits in that time span. Overall, these findings are consistent with some previous studies (such as some of those reviewed in Babikian et al., [7]) in demonstrating the disruptive impact that pediatric TBI can have on family functioning as a whole. This may be related, at least in part, to the numerous functional, cognitive, and social difficulties experienced by the child with TBI [3,4,6], as well as to the stress and emotional difficulties often experienced by caregivers [7,8]. The rates of change estimated from this sample suggest that families impacted by pediatric TBI may have difficulty regaining the functioning they lost in comparison to healthy control families; however, additional research is needed to follow these families for a longer duration post-injury (i.e., greater than one year), in order to empirically study this possibility.

## 5. Limitations and Future Directions

A number of important limitations should be taken into account when interpreting the findings from the current study. First, no measures of baseline or premorbid functioning were available, precluding the examination of pre-injury differences in family functioning or the inclusion of these measures in analyses as covariates or additional time points. As these are primarily descriptive results and are not based on true experimental methods, it would be valuable for future research to examine how family dynamics may differ (or not) between these groups after a family intervention program following pediatric TBI. Further, the current study followed participants for only one year post-TBI. A longer follow-up period would provide information as to the longer-term trajectories of family functioning in these groups, and could determine if families of children with TBI return to normal functioning, as has been examined in some other groups experiencing serious accidents or disability [27,28]. Moreover, a study of additional factors that could potentially act as moderators of these trajectories would also be valuable. Individual- or family-level variables, such as caregiver resilience or the family’s ability to afford professional in-home care, might affect the family’s ability to successfully adjust after pediatric TBI. Similarly, child or caregiver age could also be associated with family functioning in the context of TBI. It could be, for example, that families may have greater difficulty adjusting in cases of older children who functioned more independently pre-injury.

Second, the control group was selected by teachers at the educational center where recruitment occurred. As a result of recruitment limitations, random selection was not possible, and subjects were not always able to be matched on all demographic variables. Although in the current sample children with a TBI did not differ from healthy controls on sex, age, and education level, parents of healthy control children were significantly more likely to be male, have higher education levels, and higher income levels. Thus, we cannot definitively rule out the possibility that these results were influenced by group differences. It could be that, in two-parent families where a child sustains a TBI that requires caregiving, the parent with less education and earning potential might provide care at home, so that the higher-earning parent can continue to work. This might partially explain why parents of children with a TBI who completed the survey on behalf of the family were more likely to be female and to have lower education and income. In contrast, both parents of healthy control children may have been able to work, and participation in the study may have been somewhat more evenly distributed between parents, although participants in this group were also more likely to be female than male. Future prospective studies, such as those employing cohort methods in a large sample, could more directly examine the possible influence of parental sex, education, and income level on family dynamics within the context of pediatric TBI.

Currently, there is a lack of rehabilitation services for children with TBI in Latin America. For this reason, it is important to note that children with TBI who participated in this study did not receive any type of psychosocial or neuropsychological rehabilitation, nor did their parents. Thus, these results must be interpreted in the light of this context, since it is possible that the patterns of family functioning found in this study could differ from those found in other countries where comprehensive rehabilitation services are provided to these children with TBI and their families.

Finally, due to resource limitations, data on family functioning at 12 months post-injury were not able to be collected from 15 of the families of children with TBI and their matched control families. Although these groups did not differ on measures of functioning from families who were followed for all three time points, and full information maximum likelihood estimation was used to account for missing data, these missing data represent a study limitation as estimates of functioning at 12 months post-injury are based on a smaller number of families. Future research could rectify this by replicating this study in a larger sample in order to obtain more reliable and generalizable estimates of functioning.

## 6. Implications

Overall, the current study adds to the literature in demonstrating the burden that chronic conditions such as TBI can play in impacting not only the lives of the people who experience them, but also their family members, many of whom function as informal caregivers. These findings suggest that families of children with a TBI experience problems in family functioning for at least a year following the injury, in comparison to families without this concern. This study is notable in demonstrating that families of children with TBI in the current sample did not appear to catch up to healthy families, and, in some measures of functioning, actually appeared to worsen slightly.

Within a rehabilitation context, family-level intervention programs are needed to help caregivers and family members learn to adapt to their new circumstances, and to target family functioning in order to improve these relationships in the context of pediatric TBI. For example, evidence-based studies of family therapy in the context of pediatric TBI, particularly in Latin American families, have the potential to provide psychoeducation and skills training to family members to navigate the challenges and difficulties in family functioning that they may experience in the months and years following the injury. In addition, research is needed on efforts to disseminate these interventions, particularly to families who are at higher risk of experiencing family-level problems, and to those who may have limited resources for coping with pediatric TBI.

These, along with other previous findings, highlight some of the many difficulties that can be experienced by parents and other family members in the context of chronic conditions. Moreover, although the measures in the current study were reported by the parents of children with TBI and healthy controls, the scores reflect the parents’ perceptions of the functioning of the family unit as a whole. These findings help to illustrate the interpersonal nature of the effects of chronic conditions. We argue that many important outcomes, such as family and relationship functioning, social embeddedness, and emotional health and well-being, should be studied within a broader social context, as they are likely to have widespread consequences for parents, caregivers, and other family members, as well as for the individuals experiencing the chronic condition.

## 7. Conclusions

The current study investigated family satisfaction, communication, cohesion, and flexibility among families of children with TBI during the first year post-injury in comparison to same-age healthy controls in Mexico. Parents of children with a TBI reported significantly worse family functioning at each of the three time points than parents and caregivers of children without a TBI. Moreover, linear estimates of rates of change suggest that families experiencing pediatric TBI were unable to attain the levels of functioning of healthy controls during the time span studied, suggesting that these families are likely to experience long-term reductions in family functioning. Additional efforts are needed to introduce family-level interventions targeted at improving family functioning, particularly in low SES families and families with few coping resources, and more broadly, in families, like those in Mexico and Latin America, whose cultural values, family structure, and norms around caregiving for family members may put them at increased risk for these concerns.

## Figures and Tables

**Table 1:** Demographics by Group.

Variable	TBI Group *n* = 46	Control Group *n* = 46	*p*-Value
Child			
Age	10.48 (2.7)	10.67 (2.58)	0.490
Sex			0.647
Male	31 (67.4%)	34 (73.9%)	
Female	15 (32.6%	12 (26.1%)	
Number of years of education	4.59 (2.1)	5.02 (2.06)	0.049
Parent			
Age	40.00 (8.1)	37.41 (5.79)	0.077
Sex			0.045
Male	6 (13.0%)	15 (32.6%)	
Female	40 (87.0%)	31 (67.4%)	
Number of years of education	6.67 (3.7)	12.35 (2.7)	<0.001
Monthly income			<0.001
Below minimum wage	3 (6.5%)	1 (2.2%)	
1× minimum wage	10 (21.7%)	0 (0%)	
1–2× minimum wage	20 (43.5%)	11 (23.9%)	
2–3× minimum wage	9 (19.6%)	17 (37.0%)	
3–4× minimum wage	1 (2.2%)	11 (23.9%)	
4–5× minimum wage	1 (2.2%)	5 (10.9%)	
Above 5× minimum wage	2 (4.3%)	1 (2.2%)	

**Table 2:** Means and Standard Deviations of Family Variables by Group.

Variable	TBI Group	Control Group
	*M* (*SD*)	*M* (*SD*)
Satisfaction	38.27 (7.81)	45.24 (4.56)
Communication	38.38 (6.36)	45.86 (3.63)
Cohesion	27.20 (3.92)	30.45 (2.33)
Flexibility	25.91 (4.50)	32.02 (3.16)

**Table 3:** Multilevel Growth Curve Modeling Results for Family Dynamics Variables.

Effect	Satisfaction	Communication	Cohesion	Flexibility
Intercept	41.21 (0.81) ***	37.93 (0.66) ***	26.60 (0.44) ***	25.39 (0.55) ***
Group differences at Time 3	5.27 (1.15) ***	9.11 (0.93) ***	4.69 (0.62) ***	6.59 (0.78) ***
Rate of change	2.72 (0.61) ***	−0.47 (0.54)	−0.58 (0.38)	0.59 (0.46)
Group differences in rate of change	−1.63 (0.87)	1.64 (0.76) *	1.26 (0.53) *	0.47 (0.65)

Note. * *p* < 0.05, *** *p* < 0.001.

## Copyright Statement

Since Grace B. McKee and Paul B. Perrin are employees of the U.S. Government and contributed to the manuscript “Longitudinal Growth Curve Comparisons of Family Dynamics between Families Experiencing Acute Pediatric TBI and Healthy Control Families in Mexico” as part of their official duties, the work is not subject to US copyright.

## References

[1] Dewan M.C., Mummareddy N., Wellons J.C., Bonfield C.M. (2016). Epidemiology of Global Pediatric Traumatic Brain Injury: Qualitative Review. World Neurosurg..

[2] Hankinson T.C., Beauchamp K. (2016). Pediatric Traumatic Brain Injury: The Global View. World Neurosurg..

[3] Babcock L., Byczkowski T., Wade S.L., Ho M., Mookerjee S., Bazarian J.J. (2013). Predicting Postconcussion Syndrome After Mild Traumatic Brain Injury in Children and Adolescents Who Present to the Emergency Department. JAMA Pediatr..

[4] Garcia D., Hungerford G.M., Bagner D.M. (2015). Topical Review: Negative Behavioral and Cognitive Outcomes Following Traumatic Brain Injury in Early Childhood. J. Pediatr. Psychol..

[5] Bloom D.R., Levin H.S., Ewing-Cobbs L., Saunders A.E., Song J., Fletcher J.M., Kowatch R.A. (2001). Lifetime and Novel Psychiatric Disorders After Pediatric Traumatic Brain Injury. J. Am. Acad. Child Adolesc. Psychiatry.

[6] Rosema S., Crowe L., Anderson V. (2012). Social Function in Children and Adolescents after Traumatic Brain Injury: A Systematic Review 1989–2011. J. Neurotrauma.

[7] Babikian T., Merkley T., Savage R.C., Giza C.C., Levin H. (2015). Chronic Aspects of Pediatric Traumatic Brain Injury: Review of the Literature. J. Neurotrauma.

[8] Wade S.L., Taylor H.G., Drotar D., Stancin T., Yeates K.O. (1998). Family Burden and Adaptation During the Initial Year After Traumatic Brain Injury in Children. Pediatrics.

[9] Rivara J.B., Jaffe K.M., Polissar N.L., Fay G.C., Liao S., Martin K.M. (1996). Predictors of family functioning and change 3 years after traumatic brain injury in children. Arch. Phys. Med. Rehabil..

[10] Trenchard S.O., Rust S., Bunton P. (2013). A systematic review of psychosocial outcomes within 2 years of paediatric traumatic brain injury in a school-aged population. Brain Inj..

[11] Chapman L.A., Wade S.L., Walz N.C., Taylor H.G., Stancin T., Yeates K.O. (2010). Clinically significant behavior problems during the initial 18 months following early childhood traumatic brain injury. Rehabil. Psychol..

[12] Kurowski B.G., Taylor H.G., Yeates K.O., Walz N.C., Stancin T., Wade S.L. (2011). Caregiver Ratings of Long-term Executive Dysfunction and Attention Problems After Early Childhood Traumatic Brain Injury: Family Functioning Is Important. PM&R.

[13] Taylor H.G., Yeates K.O., Wade S.L., Drotar D., Stancin T., Burant C. (2001). Bidirectional child–family influences on outcomes of traumatic brain injury in children. J. Int. Neuropsychol. Soc..

[14] Raj S.P., Wade S.L., Cassedy A., Taylor H.G., Stancin T., Brown T.M., Kirkwood M.W. (2013). Parent Psychological Functioning and Communication Predict Externalizing Behavior Problems After Pediatric Traumatic Brain Injury. J. Pediatr. Psychol..

[15] Peterson R.L., Kirkwood M.W., Taylor H.G., Stancin T., Brown T.M., Wade S.L. (2013). Adolescents’ Internalizing Problems Following Traumatic Brain Injury Are Related to Parents’ Psychiatric Symptoms. J. Head Trauma Rehabil..

[16] Rashid M., Goez H.R., Mabood N., Damanhoury S., Yager J.Y., Joyce A.S., Newton A.S. (2014). The impact of pediatric traumatic brain injury (TBI) on family functioning: A systematic review. J. Pediatr. Rehabil. Med..

[17] Youngblut J.M., Brooten D. (2005). Pediatric Head Trauma: Parent, Parent–Child, and Family Functioning 2 Weeks After Hospital Discharge. J. Pediatr. Psychol..

[18] Youngblut J.M., Brooten D. (2008). Mother??s Mental Health, Mother-Child Relationship, and Family Functioning 3 Months After a Preschooler??s Head Injury. J. Head Trauma Rehabil..

[19] Lehan T.J., Stevens L.F., Arango-Lasprilla J.C., Sosa D.M.D., Jove I.G.E. (2012). Balancing act: The influence of adaptability and cohesion on satisfaction and communication in families facing TBI in Mexico. NeuroRehabilitation.

[20] Perrin P.B., Stevens L.F., Sutter M.E., Hubbard R., Sosa D.M.D., Jove I.G.E., Arango-Lasprilla J.C. (2013). Exploring the Connections Between Traumatic Brain Injury Caregiver Mental Health and Family Dynamics in Mexico City, Mexico. PM&R.

[21] Garzón A. (1998). Familismo y creencias políticas. Psicol. Política.

[22] Olson D. (2011). FACES IV and the Circumplex Model: Validation Study. J. Marital. Fam. Ther..

[23] Olson D.H., Sprenkle D.H., Russell C.S. (1979). Circumplex Model of Marital and Family Systems: I. Cohesion and Adaptability Dimensions, Family Types, and Clinical Applications. Fam. Process..

[24] Grimm K.J., Ram N., Estabrook R. (2017). Growth Modeling: Structural Equation and Multilevel Modeling Approaches.

[25] Raudenbush S.W., Bryk A.S. (2002). Hierarchical Linear Models: Applications and Data Analysis Methods. Advanced Quantitative Techniques in the Social Sciences.

[26] Cariello A.N., Perrin P.B., Rodríguez-Agudelo Y., Plaza S.L.O., Quijano-Martinez M.C., Arango-Lasprilla J.C. (2020). A Multi-Site Study of Traumatic Brain Injury in Mexico and Colombia: Longitudinal Mediational and Cross-Lagged Models of Family Dynamics, Coping, and Health-Related Quality of Life. Int. J. Environ. Res. Public Health.

[27] Boyce C.J., Wood A.M. (2011). Personality Prior to Disability Determines Adaptation. Psychol. Sci..

[28] Brickman P., Coates D., Janoff-Bulman R. (1978). Lottery winners and accident victims: Is happiness relative?. J. Pers. Soc. Psychol..

